# High-throughput single-cell whole-genome amplification through centrifugal emulsification and eMDA

**DOI:** 10.1038/s42003-019-0401-y

**Published:** 2019-04-29

**Authors:** Yusi Fu, Fangli Zhang, Xiannian Zhang, Junlong Yin, Meijie Du, Mengcheng Jiang, Lu Liu, Jie Li, Yanyi Huang, Jianbin Wang

**Affiliations:** 1 Beijing Advanced Innovation Center for Genomics (ICG), Biomedical Pioneering Innovation Center (BIOPIC), School of Life Sciences, College of Engineering, and Peking-Tsinghua Center for Life Sciences, Peking University, Beijing, 100871 China; 2 School of Life Sciences, and Tsinghua-Peking Center for Life Sciences, Tsinghua University, Beijing, 100084 China

**Keywords:** DNA sequencing, DNA

## Abstract

Single-cell whole-genome sequencing (scWGS) is mainly used to probe intercellular genomic variations, focusing on the copy number variations or alterations and the single-nucleotide variations (SNVs) occurring within single cells. Single-cell whole-genome amplification (scWGA) needs to be applied before scWGS but is challenging due to the low copy number of DNA. Besides, many genomic variations are rare within a population of cells, so the throughput of currently available scWGA methods is far from satisfactory. Here, we integrate a one-step micro-capillary array (MiCA)-based centrifugal droplet generation technique with emulsion multiple displacement amplification (eMDA) and demonstrate a high-throughput scWGA method, MiCA-eMDA. MiCA-eMDA increases the single-run throughput of scWGA to a few dozen, and enables the assessment of copy number variations and alterations at 50-kb resolution. Downstream target enrichment further enables the detection of SNVs with 20% allele drop-out.

## Abstract (web summary)

Yusi Fu, Fangli Zhang et al. integrate a one-step microcapillary array with emulsion multiple displacement amplification to obtain a high-throughput single-cell whole-genome amplification method. Their method enables copy number assessment at a resolution of 50 kb.

## Introduction

In the last decade, we have witnessed many exciting advances in single-cell studies, primarily due to high-throughput DNA sequencing technologies such as next-generation sequencing^1^. This has become the default choice to dissect complex systems, such as the trajectory of cancer evolution^2–5^ or embryonic development^6–8^, through sequencing every single cell’s transcriptome^9–13^, genome^14–19^ or epigenome^20–23^. Such omics data not only provide a comprehensive atlas for a biological system consisting of many cells with various types and states, but also offer many opportunities for new discoveries in biology and medicine.

Quantitative and precise description of the genomic variations in a heterogeneous biological system remains a challenge, primarily due to the lack of a high-throughput single-cell whole-genome amplification (scWGA) technology. First, the single cell’s genomic DNA, merely 6 pg in total for a diploid human cell, needs to be amplified a few hundred or more times in order to generate enough material to prepare a library to feed the sequencers^24^. Hence, scWGA has to be efficient. Besides, each cell can only be amplified once, so the scWGA process has to cover as much of the genome as possible^25^. Second, single-cell whole-genome sequencing (scWGS) provides two of the most important types of information regarding genomic variation, copy number variations (CNVs) and single-nucleotide variations (SNVs), for each cell. Hence, the scWGA process needs to be faithful and unbiased to preserve the information on base composition and copy numbers. Third, to capture the heterogeneity between cells, this amplification process needs to be scalable to many cells with high reproducibility.

High uniformity, low error rate and broad coverage are three major prerequisites of WGA to accurately, precisely and completely identify both CNV and SNV events of a single cell^26^. However, currently available methods still face various difficulties to completely fulfil these requirements. Two prevalent methods are degenerate oligonucleotide-primed PCR (DOP-PCR)^27^ and multiple displacement amplification (MDA)^28^. DOP-PCR has been demonstrated to be a reliable technology to provide low-noise CNV profiles. However, the genomic coverage by DOP-PCR is relatively low, limiting its applications in SNV-related studies. MDA, in contrast, exhibits satisfactory genomic coverage (~70% for a single human diploid cell), but its extremely high amplification bias prevents it from being used for high-resolution CNV calling. Two other recently established methods, multiple annealing and looping-based amplification (MALBAC)^15^ and linear amplification via transposon insertion (LIANTI)^29^, incorporate quasi-linear or linear amplification steps into the process. They can thus suppress unevenness of the amplification and obtain high coverage of a single cell’s genome; however, the whole amplification process is laborious and requires specialized reagent like custom transposons.

We recently demonstrated an alternative approach of scWGA by implementing the reaction in water-in-oil emulsion^30^. When an MDA reaction, with a volume of dozens of microliters, was evenly compartmented into a large number of picoliter droplets, the evenness of amplification could be improved while preserving MDA’s ease of operation, high fidelity and high coverage. This emulsion MDA (eMDA) approach required monodispersed emulsion to ensure uniform amplification. We and others have shown two ways of generating these droplets, using either microfluidic chips^30^ or spinning capillary in oil^31^. However, neither of these approaches was easy to operate without specific training or commercially available instruments, nor would they be compatible with the operations with which biomedical researchers are familiar. Moreover, such emulsion generation methods are difficult to scale to higher throughput. To overcome these difficulties, we can apply our recently published droplet generation method based on micro-capillary array (MiCA)^32^ to generate water-in-oil emulsions with high speed, great monodispersity, zero sample loss and compatibility with general laboratory supplies.

In this study, we combined MiCA emulsion generation with eMDA (MiCA-eMDA), achieving high throughput and overcoming the technical barriers for laboratories with limited microfluidics experience. We demonstrated that the centrifuge-driven MiCA emulsion generation was naturally high-throughput, with the capacity to simultaneously process up to 48 samples in a single centrifugal run. This simple and efficient emulsification strategy can ultimately facilitate biological applications that utilize droplets. We have proven that, with appropriate oil and surfactant combinations, such MiCA-generated emulsion had no influence on the efficiency of amplification compared with the previously reported microfluidic eMDA results. We also applied hybridization-based target enrichment on our MiCA-eMDA products, enabling the simultaneous identification of both CNV and SNV from the same single cells. We processed 46 single cells with MiCA-eMDA and obtained the CNV profile through shallow WGS. Single-cell analysis revealed a 10-Mb heterogeneous CNV otherwise buried in the bulk results. We further performed targeted deep sequencing on 15 cells and detected SNV with 20% allele drop-out.

## Results

### High-throughput emulsion generation and whole-genome amplification

**Fig. 1:**
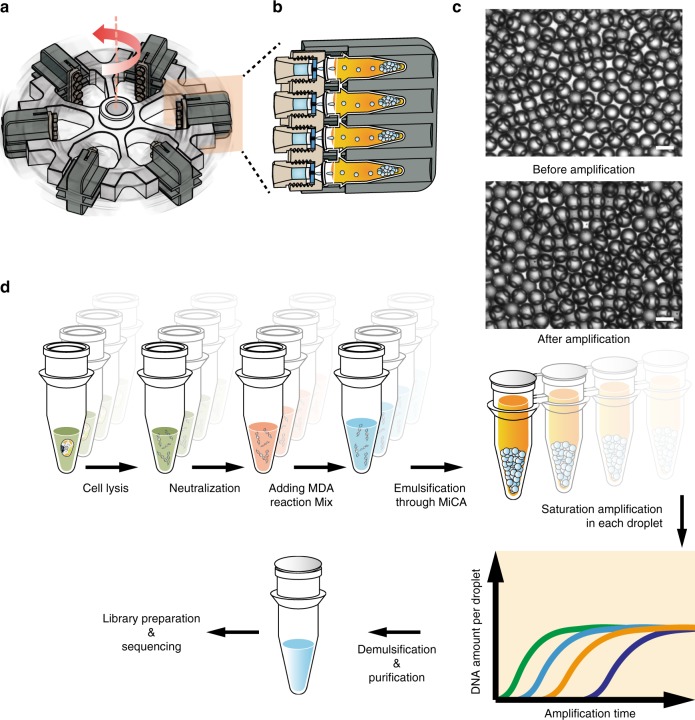
Overview of high-throughput emulsion whole-genome amplification. **a** The design of rotor and swing buckets for high-throughput centrifugation. **b** The cross-section view of one swing bucket. **c** The droplets are stable during the whole amplification process. (Scale bar: 50 μm). **d** High-throughput eWGA consists of cell lysis, neutralization, addition of reaction mix and high-throughput droplet generation through centrifuge

In our previous study on MiCA droplet generation, this approach was demonstrated in a low-throughout fashion using a standard swing bucket rotor^32^. Here, we re-designed the swing buckets (Supplementary Fig. 1) to further improve the throughput. With six four-tube buckets in a rotor, the emulsification throughput was increased to 24 samples per run (Fig. 1a). During centrifugation, the aqueous reaction mixture containing single-cell lysate, primers, dNTPs and phi-29 polymerase was spun through MiCA at >15,000 × *g* (Fig. 1b) and formed 40-µm-diameter droplets in the oil phase composed of 93% isopropyl palmitate and 7% ABIL EM180 (Supplementary Fig. 2). This process of emulsion generation is extremely efficient, with a rate of droplet production of over 2000 per second. When using a seven-hole MiCA plate, it typically took less than 8 min to spin down each sample, producing more than 10^6^ droplets.

Cell lysis was implemented by manually picking up and placing each single cell into 2 μL of PBS buffer, followed by the addition of 1.5 μL of alkaline cell lysis buffer and 10 min of incubation at 65 °C to release the genomic DNA. Then 1.5 μL of neutralization buffer was added to each microtube to terminate the lysis step. Subsequently, amplification mix containing all of the necessary MDA reagents was added. This entire reaction mix (10–100 μL) was then emulsified using MiCA through centrifugation. We performed a systematic combinatorial test on the surfactant recipe and selected 7% ABIL EM 180 to stabilize the isopropyl palmitate oil phase. The emulsion was incubated at 30 °C for 8 h, before heat inactivation of the phi-29 polymerase at 65 °C. The droplets maintained monodispersity throughout the whole process (Fig. 1c). Our previous test suggested that extending the reaction time beyond 8 h would not confer additional benefits to the eMDA process. The reactions were terminated by heating and isobutanol was added to demulsify the water-in-oil droplets. Then, purification was performed with Zymo-Spin™ columns (Zymo Research) coupling with DNA Clean & Concentrator kit (Zymo Research) following the recommended protocol. After demulsification and purification, we usually recover ~1 µg of high-molecular-weight amplification product, which is more than enough for downstream sequencing library preparation (Fig. 1d). The whole process of MiCA-eMDA is simple, making it possible for a single researcher to complete dozens of scWGA procedures and to construct corresponding libraries within a day or two.

### A web-facilitated analysis pipeline for single-cell genomics

**Fig. 2:**
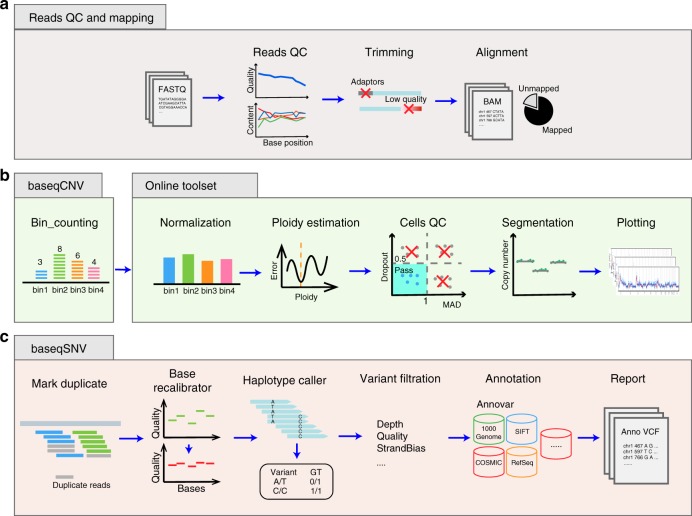
The web-facilitated analysis pipeline. **a** The sequencing reads first undergo quality control and filtered reads are mapped to reference genome. **b** The CNV pipeline includes baseqCNV generating sequencing depth file and the online toolset for downstream analysis and visualization. **c** The baseqSNV package for single-cell SNV detection

Quantitative analysis of single-cell sequencing data is difficult, especially when such data have to be obtained through high-gain amplification. When handling small datasets from only a few single cells, it is common to manually check the result of each single cell. Processing large datasets, however, requires more efficient strategies. Therefore, we established an analysis pipeline (Fig. 2) that automatically implements the whole process required for single-cell genomic analysis. This pipeline first performs quality control of the raw sequencing data and aligns the filtered reads to the reference genome (Fig. 2a). Then, the pipeline provides two different analytical functions, baseqCNV for CNV analysis with low-coverage WGS data (Fig. 2b) and baseqSNV for SNV identification with targeted deep sequencing data (Fig. 2c). BaseqCNV and baseqSNV are Python-based packages and are easy to install and configure. With raw sequencing data input in fastq format, these two packages can automatically process the data and generate the files needed for visualization. The entire procedure is user-friendly, including for those with limited bioinformatics experience.

For CNV analysis, single dynamic binning of the genome is required. BaseqCNV counts the reads in each bin of the genome and the outputs can be submitted to an online toolset (http://wgs.beiseq.cn) for downstream analysis. After correcting the sequencing depth based on GC content, the online toolset calculates the ploidy of nondiploid cells using our absolute copy-number determine (ACD) algorithm and determines the copy number of each bin with circular binary segmentation. We use median absolute deviation (MAD) as a metric to evaluate the evenness of amplification. The dropout ratio, calculated as the proportion of bins with zero aligned reads, indicates the genome coverage. Low-quality cells can be filtered out using these two metrics. Neighbouring bins can be merged to segments before final visualization. BaseqSNV follows the GATK best practice for variant calling^33^ (Fig. 2c). Allele dropout rate and coverage breadth are calculated using unamplified bulk samples as a control. We have uploaded the data described in this paper for users to explore and reproduce the results using the packages (see Supplementary Information for details).

### High-throughput single-cell MiCA-eMDA and CNV analysis

The improved evenness by eMDA is derived from the compartmentalization. Within each droplet, the amplification is independent, which reduces competition and bias. The number of compartments is critical to the final performance of eMDA, markedly affecting the evenness of amplification and the mapping ratio of the sequencing reads. Once the droplet size is fixed, the number of droplets is simply determined by the volume of the aqueous reaction mixture. With single HeLa cells as starting material, we constructed a series of reactions ranging from 10 to 100 μL in volume, and compared the results to those from the conventional MDA performed in a microtube (tube-MDA) as well as from the emulsion generated by microfluidic chips (chip-eMDA). Before sequencing, we calculated the amplification yield by quantitative PCR. The results showed no difference among the different reactions (Supplementary Fig. 3, Supplementary Table 1).

**Fig. 3:**
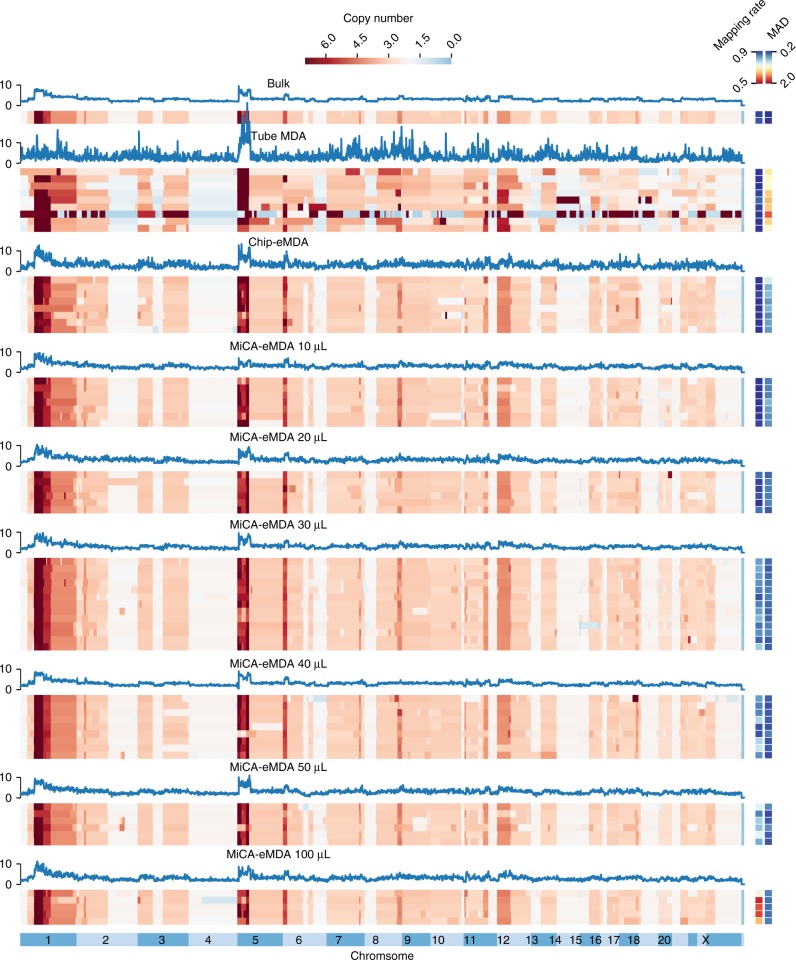
Whole genome CNV distribution of different WGA method amplified single cell samples compared to unamplified sample. Heatmap representing the whole genome CNV distribution of 1 M bin-size for each sample. A representative CNV pattern plot for each method is also shown

We then conducted shallow whole-genome sequencing at an average depth of 1.3x per cell (Supplementary Table 2) for all of the 63 single-cell WGA products. We also included two unamplified bulk samples using purified genomic DNA from 107 HeLa cells for comparison. The baseqCNV pipeline can automatically process scWGS data and generate representative whole-genome CNV profiles for each single cell at various resolutions (Fig. 3, Supplementary Data 1, Supplementary Data 2 and Supplementary Fig. 4). Most of the samples showed similar mapping ratios, typically above 50%, while the amplification uniformity was quite diverse among the amplification methods.

**Fig. 4:**
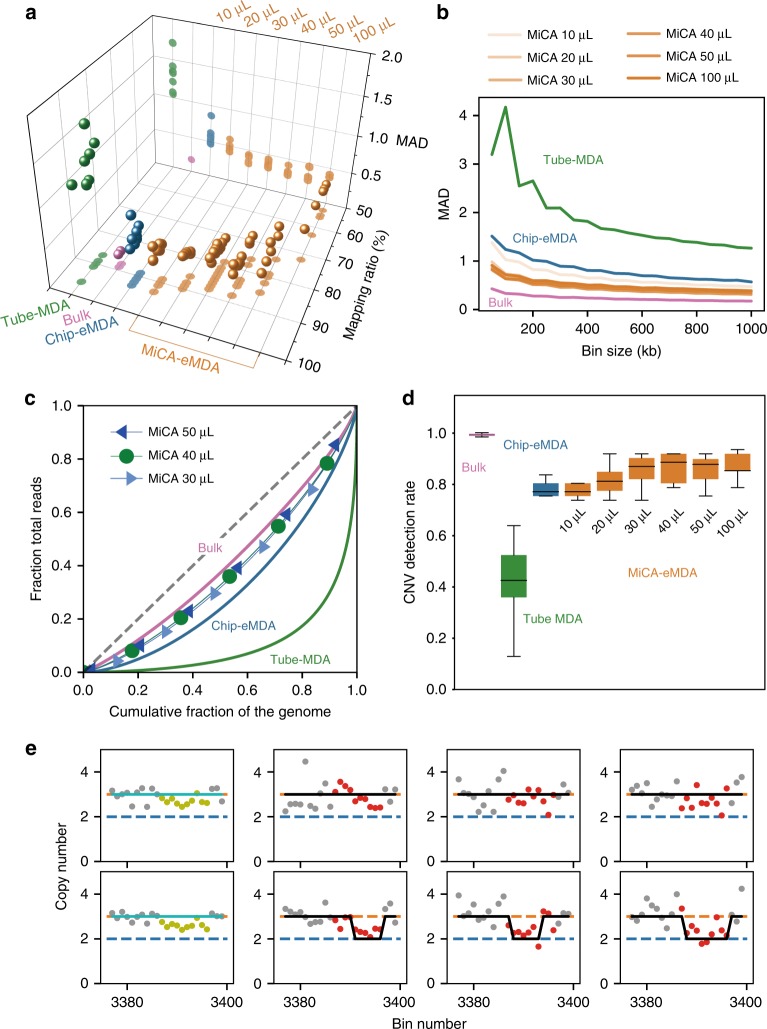
The comparison of copy number detection ability between conventional MDA, on-chip eMDA and high-throughput MiCA eMDA. **a** The MAD and mapping rate for different amplification method. MAD decrease and then increase with enlarging reaction volume, mapping rate dropped with larger reaction volume. **b** MAD decrease with large bin size on a whole, MiCA are evener than chip-eMDA for all the bin-size tested. **c** The Lorenz curves of coverage uniformity for single cells amplified by different method compared with unamplified genomic DNA. **d** The detection rate of copy number change in single HeLa cell compared to unamplified sample. **e** A region with mean copy number of 2.67 in unamplified sample(two samples on the left), while a 2–3 distribution within single cell samples. Red line showing copy number of 3 and blue line showing copy number of 2, red dots represent the heterogenous region in single cell and yellow for unamplified sample. Black line is the integer copy number determined in single cell and cyan for unamplified sample

Using the bulk sample as a reference, all of the single-cell samples amplified by eMDA approaches (chip-eWGA and MiCA-eWGA) showed similar CNV profiles. In contrast, the single-cell samples amplified by conventional ‘one-pot’ tube-MDA showed extraordinarily high bias randomly distributed across the whole genome. Such bias is a major obstacle to confidently determining the copy numbers with high resolution. Quantitatively, the 1-Mb bin MAD value of each eMDA sample was generally between 0.30 and 0.47, while the conventional tube-MDA has a typical MAD value of 1.3 (Fig. 4a, Supplementary Data 3). It is also worth pointing out that the MAD values are based on the absolute difference between adjacent bins. Therefore, aneuploidy or large CNVs would not erroneously increase the MAD value. MAD value is also dependent on bin size. For a given sequencing dataset, a larger bin size will give a smaller MAD value, which is related to sampling noise. We tested different bin sizes from 50 kb to 1 Mb, and confirmed that MiCA-eMDA products exhibited the lowest MAD values and that conventional tube-MDA had the highest MAD values, representing high evenness of amplification of MiCA-eMDA products (Fig. 4b, Supplementary Data 3).

As expected, reaction volume affects amplification uniformity through compartment number, as quantified by MAD values. When droplet size was kept constant, a large volume (high compartment number) and a small volume (low compartment number) both resulted in elevated MAD values and there was an optimal volume between the two. We obtained the lowest MAD value in the serial experiments from 40-μl reactions, which corresponded to 1.3 × 10^6^ 40-μm-diameter droplets. Given the fact that genomic DNA is usually fragmented to about 10–20 kb by our laboratory operations, the genome of a single diploid cell is broken into ~1 million single-strand DNA fragments. Thus, most droplets will not contain more than one template. If the number of droplets is too low, each droplet will take too many DNA fragments. Amplification bias among these fragments is inevitable and the MAD value will be high. Alternatively, if the number of droplets is too high, there will be many droplets with no DNA fragments but lots of random primers. Such droplets will still produce a large number of products through random primer annealing. These products cannot be mapped to the genome, leading to a low mapping ratio and a waste of sequencing effort, and thus require a higher sequencing depth. In our experiments, we observed such monotonically decreasing mapping rate with an increase of reaction volume (Fig. 4a). Based on the above-mentioned results, we concluded that the most suitable reaction volume for our MiCA-eMDA approach, with the optimal emulsification conditions to generate 40-μm droplets, is between 30 and 50 μL, which corresponds to DNA fragments in each droplet numbering from 0.4 to 0.7 for a 10-kb DNA fragment size.

Besides amplification bias reflected by MAD values, a more comprehensive assessment of the evenness of amplification can be obtained by plotting the Lorenz curves of coverage for each sample (Fig. 4c, Supplementary Data 4). A perfectly uniform distribution of sequencing reads across the genome would create a diagonal line in the Lorenz plot, and any biased distribution would present a curve towards the edges away from the diagonal. The Lorenz curve of an unamplified bulk sample would clearly be very close to the diagonal since the only bias was introduced by the library preparation and sequencing process, which have been optimized over the years. The single-cell eMDA results showed Lorenz curves close to the result of bulk samples, demonstrating markedly high evenness compared with the conventional tube-MDA reactions. MiCA-eMDA further outperformed chip-eMDA, probably due to the higher monodispersity of emulsion droplets and less material loss during sample handling, which is in accordance with the MAD results. Reaction volumes ranging from 30 to 50 μL showed similar distributions of evenness.

With the CNV profiles generated by baseqCNV, we could compare the CNV detection ability of different amplification protocols/conditions. Assuming a homogeneous cell population in the bulk sample, we identified 61 CNVs ranging from 1.6 to 190 Mb (Supplementary Table 3) by rounding the calculated copy numbers to the nearest integers. These CNVs should be shared by the vast majority, if not all, of the cells. The other bulk replicate showed a high detection rate (98.4%, 60 out of 61), proving the robustness of the CNV detection by our pipeline. Next, we analysed how many of these CNVs had been detected in each scWGS dataset (Fig. 4d, Supplementary Data 3). Tube-MDA products showed low CNV detection rates (42% on average) due to high amplification bias, whereas eMDA products could faithfully retain the copy number features with detection rates between 77 and 87%. In accordance with the MAD results, the CNV detection rate reached a plateau in MiCA reactions with a volume over 30 μL.

Lower amplification bias of eMDA leads to higher accuracy of copy number determination, even for small CNV events. While analysing the CNV profile of bulk samples, we found an interesting site on chromosome 12 where the inferred copy number was 2.67. Such a fractional copy number of ensemble measurement strongly suggested cell heterogeneity in the population, which consisted of single cells with integral copy numbers. When carefully checking this 10-Mb region in the scWGS data, we clearly identified discrete copy numbers of 2 or 3, showing the expected heterogeneity among single cells (Fig. 4e, Supplementary Data 3). Such results demonstrate that high-throughput single-cell analysis is essential for heterogeneous samples, where analyses of bulk samples can only provide an average representation of the cells and the important differences among cells remain obscure.

### Single-cell SNV analysis

**Fig. 5:**
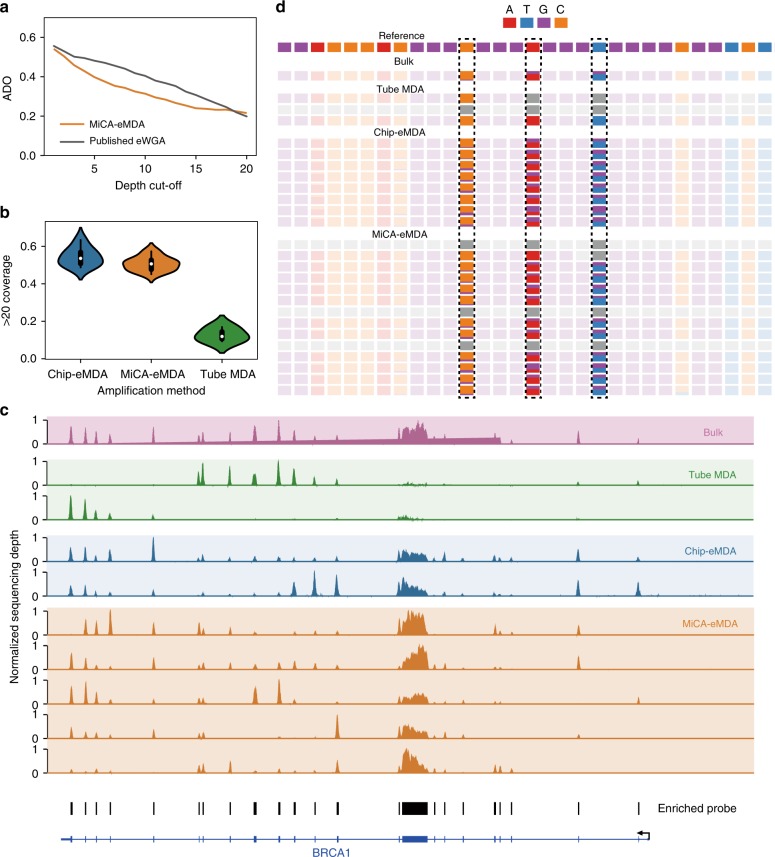
The comparison of single nucleotide detection ability between conventional MDA, on-chip eMDA and high-throughput MiCA eMDA. **a** Deep whole genome sequencing shows comparable allele drop-out result when comparing diploid region with published data. **b** High-throughput MiCA-eMDA shows a comparable coverage of region enriched compared to standard on-chip emulsion method, both are better than the conventional MDA. **c** A base resolution sequencing depth distribution for the enriched BRCA1 gene. **d** The base composition of a region containing three heterogenous SNVs

To validate the SNVs detected through scWGA by MiCA-eMDA, we performed deep sequencing of a single cell to an average depth of 48×, and compared the results with the published chip-eMDA data^30^. Since many regions of the HeLa genome are polyploid, we focused on the diploid regions for heterozygosity and allele drop-out analyses. MiCA-eMDA showed an allele drop-out rate comparable to that of the chip-eMDA approach with different sequencing depth cut-offs (Fig. 5a, Supplementary Data 5). This indicated that there was no extra fragment loss caused by the introduction of MiCA, which exhibited high genome coverage and a low false-negative rate when detecting SNVs due to fragment loss.

Whole-genome deep sequencing, however, is not practical for high-throughput single-cell SNV analysis due to the high costs. Targeted deep sequencing can better utilize the sequencing capacity. In a proof-of-concept study, we designed a gene panel covering 390 cancer-related genes (~1.3 Mbp) and performed deep sequencing on some amplification products together with unamplified bulk samples to evaluate the ability to detect SNVs. With a mean sequencing depth of over 180x, both MiCA-eMDA and chip-eMDA covered over 50% of all targeted loci with sufficient depth for SNV detection, despite coverage noise at the single-base resolution (Fig. 5b, Supplementary Data 5). We validated the fragment loss of the enrichment process and found that this loss was negligible (Supplementary Fig. 5). When we examined the coverage distribution across the entire gene body, we clearly identified more skewed coverage depth profiles in the conventional tube-MDA data (Fig. 5c, Supplementary Data 6). We then focused on heterozygous SNVs (Fig. 5d, Supplementary Data 5) identified from the unamplified bulk sample. We found comparable allele drop-out rates between MiCA- and chip-eMDA approaches (Supplementary Fig. 6), much lower than in conventional tube-MDA. Such merits of SNV identification allow high-confidence and quantitative analyses of SNV events in single cells using our high-throughput MiCA-eMDA approach (Supplementary Fig. 7).

## Discussion

While the continuous development of sequencing capacity paves the way towards high-throughput single-cell analysis, achieving comprehensive and informative descriptions of complex populations requires direct and extensive progress in single-cell technologies. There have been two major technical approaches to improving the performance of single-cell WGA.

The first approach deals with the fundamental chemistry of amplification. Two relatively conventional methods, DOP-PCR^27^ and MDA^28^, are the most popular scWGA chemistries. DOP-PCR has been proven to be excellent in controlling amplification bias, but suffers from low coverage and a high rate of amplification error when working with single cells. MDA, in contrast, has been regarded as the most efficient amplification method with the lowest error rate and high coverage breadth, but exhibits extremely large amplification bias due to the random initiation of isothermal amplification. In practice, DOP-PCR is preferred for high-resolution CNV analysis, whereas MDA is the choice for SNV analysis or chromosome-level CNV with careful normalization. Two other methods, MALBAC^15^ and LIANTI^29^, have been developed recently to provide highly even amplification for single cells via quasi-linear or linear amplification. MALBAC, however, cannot offer sufficient accuracy for SNV identification, mostly due to the low fidelity of polymerase used. LIANTI, in contrast, greatly reduces the rate of amplification error through in vitro transcription. Although LIANTI performs well in both CNV and SNV identification, the complex experimental process requires skill for its operation, thus making high-throughput implementation impractical.

The second approach, mostly based on microfluidics, focuses on reducing the reaction volume^17,34,35^. An scWGS study commonly targets tens to hundreds of cells, so it is preferred to perform each cell’s reaction in a small volume and process a large number of cells in parallel. Previous reports showed that the use of a small reaction volume might help in reducing the bias of scWGA, probably due to the suppression of preferably amplified fragments. An alternative option was to perform scWGA within a hydrogel, which increased the local concentration of the template DNA^34^. However, we found that such an approach typically resulted in low coverage across the genome when working with single mammalian cells, the genome of which is relatively large. In addition, the microfluidic devices are not easy to fabricate or to operate.

In this paper, we introduce an alternative approach, MiCA-eMDA, by combining a centrifuge-based emulsion generation technology with emulsion MDA to overcome three major challenges. First, although our previous studies demonstrated that emulsification greatly improved the MDA evenness, the chip-based emulsion generator is still an ideal choice for most biologists. In contrast, MiCA emulsification, realized by one-step rapid centrifugation, greatly simplifies the most difficult experimental process and hence further promotes eMDA as a simple scWGA protocol. Second, a centrifuge is a natural high-throughput instrument, which can simultaneously process many samples, making the scalability of MiCA-eMDA limited only by the number of centrifugal tubes that can be placed in the rotor. With a simple high-capacity swing bucket, as we demonstrated here, a common desktop centrifuge can process 24 or 48 samples with a single run in no more than 15 min. Third, in addition to the high uniformity, eMDA has also a high yield to produce a large amount of product for targeted enrichment. We have shown that integrated CNV/SNV analysis of single cells is practical and easy to operate, especially with our user-friendly pipeline.

In summary, we have developed an easy-to-use high-throughput emulsion generation device for whole-genome amplification of single cells. With a centrifuge, the whole emulsification step can be completed within 15 min and with less than 5 min of hands-on time. The diameter of the droplet is tightly distributed and stable throughout the amplification process. When MDA reaches saturation in each droplet, the original template in each droplet is amplified with similar amplitude, resulting in evenly amplified products. With sufficient amplification time and reagents, the high amplification gain would result in low fragment loss and high genome coverage. Through this device, we can boost the throughput of single-cell WGA and detect CNV and SNV inside a single cell at the same time. The amplified DNA is also compatible with target enrichment to detect CNV and SNV inside the same single cells. Researchers can focus on regions of interest and obtain SNV information at a relatively low sequencing cost. With the web tool that we provided, researchers can easily reproduce the results that we present in this paper and perform single-cell analysis of their own data. To detect the heterogeneity within a cell population, high throughput and good preservation of genomic information are essential. For diploid mammalian cells, there are only two copies for each DNA molecule, which could be amplified only once. Thus, simultaneous detection of SNV and CNV is difficult to achieve, especially when high throughput is also needed. Our MiCA-based emulsion amplification method solves this problem effectively. We here provide a comprehensive solution for single-cell genomic analysis from DNA amplification to bioinformatic analysis and output.

## Methods

### Device setup and droplets generation

Compared to our previous published paper, the swing bucket rotor is redesigned to hold a 4-Tube Strips (half of 200 μL PCR 8-Tube Strip) (Supplementary Fig. 1) to improve the throughput of droplet generation per run. Meanwhile, the manifold is also redesigned to hold 4 MiCA plates. After inserting MiCA plates into the manifold, plastic screws are used to keep the plates in place. When aqueous phase is transferred to the sample reservoir on the top of the plate and apply centrifugal force, the aqueous reaction buffer would flow through the MiCA plate and form droplets in the collection tube underneath which contains oil and detergent (93% isopropyl palmitate, 7% Abil EM180). Using this new design, one single swing bucket rotor is capable of emulsifying 4 samples in parallel. At 15,000 rcf centrifuge speed, the whole emulsification process could be completed within 20 min even for as large as 100 μL reaction buffer input. For a conventional centrifugal machine, six swing bucket rotors could be placed in parallel, thus up to 48 samples would be emulsified in a single run.

### Droplets uniformity and stability

To verify the uniformity of droplet size distribution between the four emulsified samples inside a rotor, we first use 1 × PCR buffer (NEB) as aqueous phase input for control. At 15,000 rcf centrifuge speed, the average diameters of droplets generated from four MiCA plates in a single swing bucket rotor are 38.93 μm (CV = 0.03645), 40.69 μm (CV = 0.01819), 39.01 μm (CV = 0.02675) and 39.06 μm (CV = 0.02729) (Supplementary Fig. 2A). From the size distribution we could conclude the droplets are monodispersed with diameter around 40 μm under this condition, and droplets generated from four different MiCA plates have no difference (Supplementary Fig. 2B). The stability of the droplets was confirmed by observing the droplets after amplification.

### Cell culture

HeLa cell were provided by Professor Yujie Sun in the School of Life Sciences at Peking University. We cultured the HeLa cell using DMEM medium (Invitrogen) with 10% FBS (Invitrogen) and 1%PS (Invitrogen) at 37 °C in a humidified incubator supplemented with 5% CO_2_. Cells were passaged before becoming fully confluent in order to maintain their proliferation phenotype. Cells were digested with 0.25% trypsin containing 0.1% EDTA (Invitrogen) and wash 3 times with PBS before transferred and resuspended in new petri dish.

### Single cell preparation

Cell suspension were first diluted using PBS and dispersed into single cell. Then mouth pipet was used to pick single cell from the diluted suspension under stereoscope. Then the picked single cell was washed and transferred to a 200 μL centrifuge tube containing 2 μL PBS buffer using new and clean capillary tip with minimum residue buffer from upstream process.

### Single cell MiCA-eMDA reaction

We first added 1.5 μL Buffer D2 (REPLI-g Single Cell Kit) to single cell suspension and the single cell was fully lysed after incubating at 65 °C for 10 min. The lysis was terminated by adding 1.5 μL Stop Solution to neutralize the alkaline lysis buffer. Then the released genomic DNA was fragmented and denatured by heating at 98 ℃ for 4 min. Reaction buffer (final concentration of 1× Phi 29 polymerase reaction buffer (NEB), 50 μM N6 primer (Invitrogen), 1 mM dNTP) was then mixed with the DNA and heated to 95 °C for 2 min and quick cool to 4 ℃ to maintain the single strand state of the DNA, the mixture is placed at 4 ℃ for another 20 min for random primer to fully anneal to the single stranded gDNA. Phi-29 polymerase is added right before emulsification to prevent non-emulsified amplification.

After adding Phi-29 DNA polymerase (final concentration at 0.8 units/μL, NEB), the whole reaction mix is ready for emulsification. The mixture was transferred onto the top of MiCA plate within the 4-MiCA plate holder. After applying centrifuge, the reaction mix was dispersed into separated droplets and collected in PCR 4-Tube strips (half of PCR 8-Tube Strips) containing oil and detergent underneath. Emulsification of up to 48 samples could be operated in parallel. Amplification was carried out at 30 ℃ for 8 h and terminated by heating at 65 °C for 10 min to inactivate the polymerase.

### Single cell Chip-eMDA reaction

The experiment process of chip-based emulsion MDA was described in detail in our previous published paper^30^. In summary, the whole process is the same as MiCA-eMDA except the emulsification step of the MDA reaction, home-made PDMS chip or glass chip (Dolomite) with flow-focusing feature was used to disperse the reaction mix into monodispersed droplets. The oil used was HFE-7500 containing 1% (w/w) EA surfactant (RainDance Technologies).

### Conventional single cell MDA reaction

The reaction mix was prepared the same as MiCA-eMDA reaction. After adding Phi-29 polymerase, the reactions were directly initiate by incubating the centrifuge tube at PCR machine at 30 °C. After 8 h amplification, the reaction mixture is heated at 65 °C for 10 min to terminate the amplification process.

### DNA purification, library preparation and sequencing

After reactions were terminated through heat, we used isobutanol to demulsify the water-in-oil droplets and used Zymo-Spin™ columns (Zymo Research) coupling with DNA Clean & Concentrator kit (Zymo Research) kit to purify the amplified DNA following the recommended protocol. Quality control of the amplification result was performed by measuring DNA amount using Qubit dsDNA HS Assay (Thermo Fisher Scientific) and evaluating the amplification bias through quantitate PCR (Supplementary Fig. 3, Supplementary Table 1). Sequencing libraries were built for Illumina platform using TruePrep DNA Library Prep Kit V2 for Illumina (Vazyme) using 50 ng DNA as input. The libraries were sequenced on Illumina Hiseq 4000 or Hiseq 2500 platform. The sequencing details of each library are listed at Supplementary Table 2.

### Home-made DNA panel enrichment

To improve sequencing depth without increasing cost, we designed a gene panel consisting 390 cancer-related genes. We carried out target enrichment following Agilent’s protocol (G7530-9000). Briefly, we mixed eight WGS libraries in equal ratio, followed by AMPure beads purification and concentration. We then incubated 500–750 ng DNA library mixture with 500 ng RNA probes for hybridization. We recovered the captured library molecules with MyOne Streptavidin T1 beads and amplified with PCR before sequencing.

### CNV calling

We developed a python packaged called baseqCNV (http://wgs.beiseq.cn) to process the single cell sequencing data. This package integrates multiple software and provide a full solution to analyze single cell sequencing data from alignment to CNV result visualization. Reference genome were first split into 50 k-bases bins using dynamic binning method described by Baslan et al.^36^ and dynamic bin files for Homo sapiens reference genome (hg19) and Mus musculus reference genome (mm10) were included with baseqCNV. The raw sequencing data in fastq format were mapped to the reference genome using Burrows-Wheeler Aligner (BWA)^37^ and the number of uniquely mapped reads in each bin was calculated. The bins with no uniquely mapped reads were defined as dropout. Then the read counts in each bin were corrected for GC biases using LOWESS smoothing in R, the GC biases before correction was plotted for quality control. To determine the ploidy of a single cell with copy number variation, we calculated the residual error (the square of deviations between the raw and the absolute copy number) with a series of different ploidy numbers. The one with the smallest residual error was assigned as the real ploidy, the MAD was calculated by averaging all the absolute copy number differences between two adjacent bins. MAD was used to depict the technical noise during amplification. All the cells were then subjected to a quality control step, cells were valid with MAD <= 1 and dropout ratio <= 0.5. CBS segmentation tools (DNAcopy in R) was then used to calculate an integer copy number for each bin. The normalized read counts in each bin and copy number for each cell were visualized using ggplot2 package in R.

### SNV detection

The detection follows the baseqSNV package (http://wgs.beiseq.cn). Reads were first trimmed and filtered with the following criteria: adaptors were removed according to reverse complementary sequence of the pair-end reads, and filtered reads were dynamically trimmed with a Phred cutoff of 20. Reads were then mapped to human GRCh38 reference genome by Bowtie2 (MapQ ≥ 15)^38^. The bam files are first labelled for the PCR duplications with Picard MarkDuplicate. Mapped bam was realigned by GATK IndelRealigner^33^. Genotype was called with realigned bam on target region by GATK HaplotypeCaller (BaseQ ≥ 15). Heterozygosity analysis was performed using standard of minor allele frequency ≥ 5%, depth ≥ 5 (single cell), and MAF ≥ 20%, depth ≥ 30 (unamplified sample).

### Reporting summary

Further information on experimental design is available in the Nature Research Reporting Summary linked to this article.

## Supplementary information

Description of Additional Supplementary Files

Supplementary Information

Reporting Summary

Supplementary Data 1

Supplementary Data 2

Supplementary Data 3

Supplementary Data 4

Supplementary Data 5

Supplementary Data 6

## Data Availability

Sequencing data is available at SRA under accession code SRP188831. All other data can be accessed on our website (http://wgs.beiseq.cn).

## Code availability

Users can download the baseqCNV and baseqSNV package from Python Software Foundation (www.pypi.org) for CNV and SNV analysis. The software enables the submission or upload of results for further visualization from command line (http://wgs.beiseq.cn).
