# Supplementary material for: High-throughput single-cell whole-genome amplification through centrifugal emulsification and eMDA
Source: Commun Biol. 2019 Apr 29;2:147. doi: 10.1038/s42003-019-0401-y (PMC6488574; doi:10.1038/s42003-019-0401-y)
Supplement: Supplementary file 1 — Description of Additional Supplementary Files [file 42003_2019_401_MOESM1_ESM.pdf]

Description of Additional Supplementary Files

File Name: Supplementary Data 1
Description: The raw data for the representative CNV pattern and the raw data for MAD and mapping rate for each sample in Figure 3.

File Name: Supplementary Data 2
Description: The raw data for the heatmap in Figure 3

File Name: Supplementary Data 3
Description: The raw data for Figure 4a, 4b, 4d, 4e.

File Name: Supplementary Data 4
Description: The raw data for Figure 4c.

File Name: Supplementary Data 5
Description: The raw data for Figure 5a, 5b, 5d.

File Name: Supplementary Data 6
Description: The raw data for Figure 5c
