# Supplementary material for: High-throughput single-cell whole-genome amplification through centrifugal emulsification and eMDA
Source: Commun Biol. 2019 Apr 29;2:147. doi: 10.1038/s42003-019-0401-y (PMC6488574; doi:10.1038/s42003-019-0401-y)
Supplement: Supplementary file 2 — Supplementary Information [file 42003_2019_401_MOESM2_ESM.pdf]

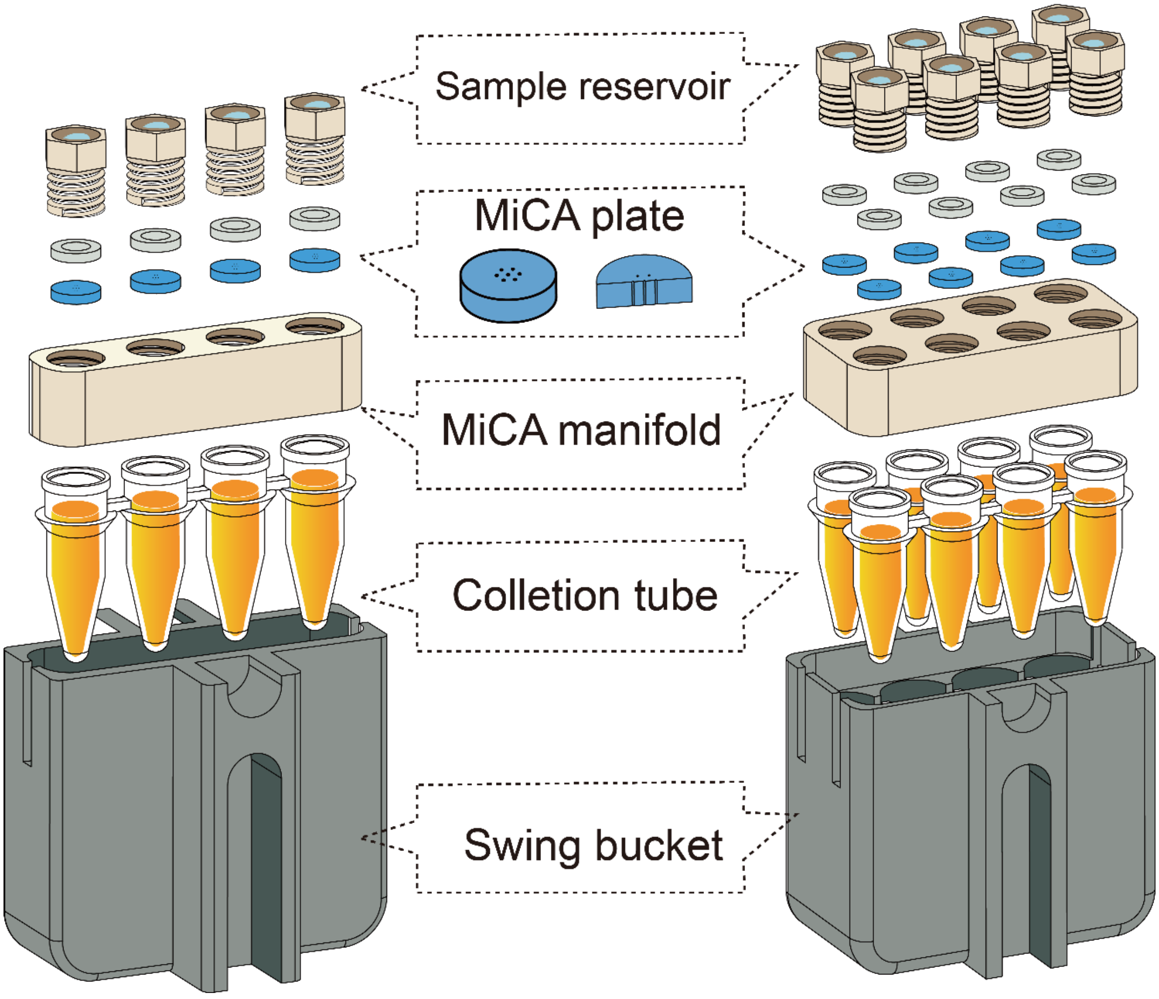

Figure S1. Schematic illustration of high-throughput centrifugation MiCA assembly (left: 24 samples per run. Right: 48 samples per run).

a)

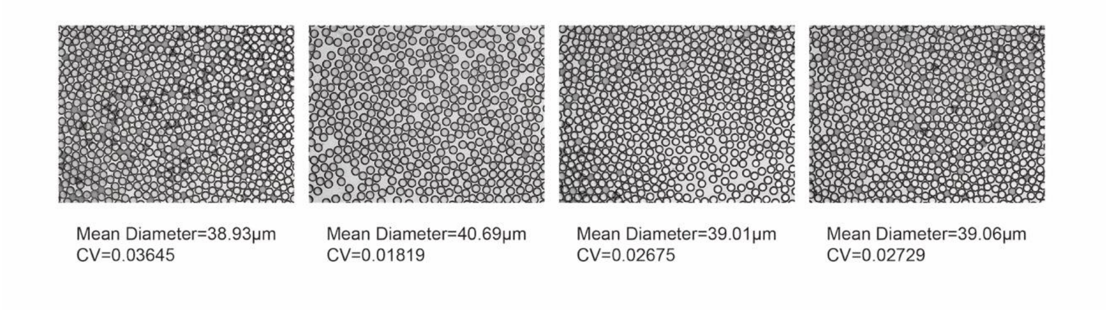

b)

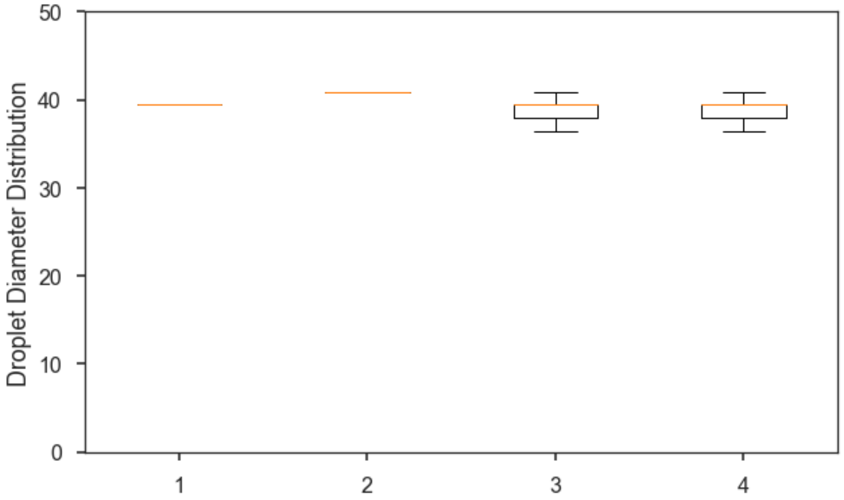

Figure S2. a) Microscopic images and b) size distribution of the water-in-oil emulsion droplets generated by four MiCA in the same single swing bucket.

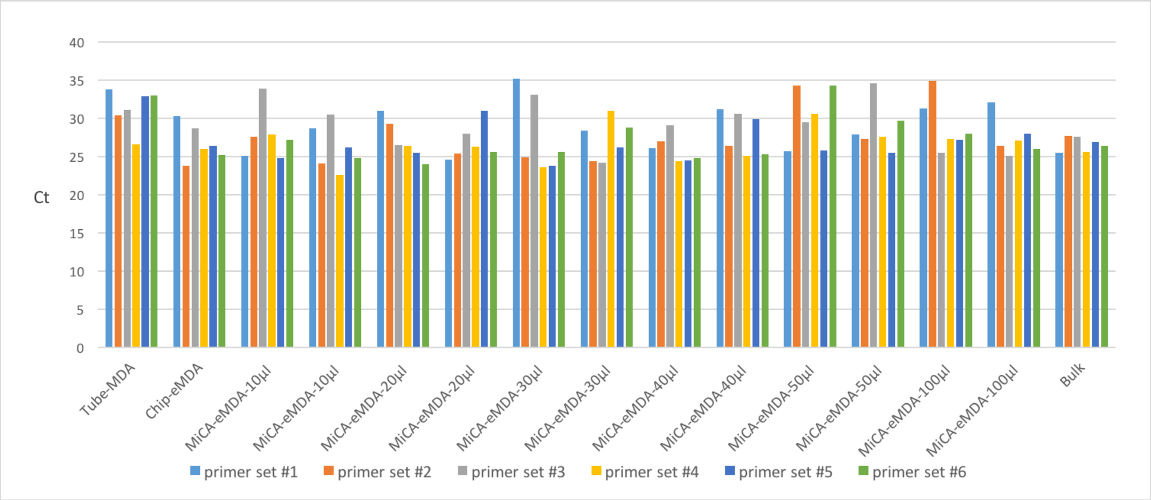

Figure S3. Quantitative PCR QC results of the WGA products.

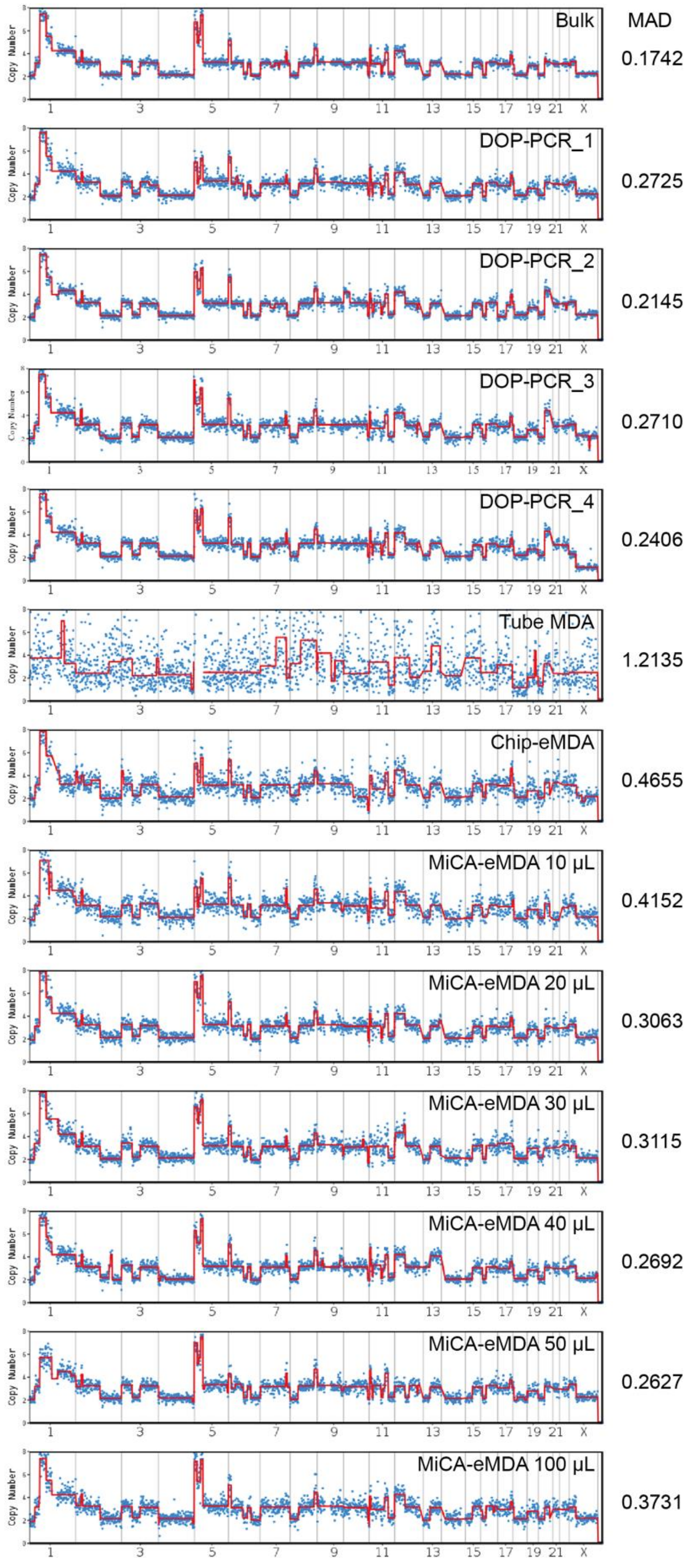

Figure S4. The whole-genome copy number profiles of different samples. (More on our website http://wgs.beiseq.cn)

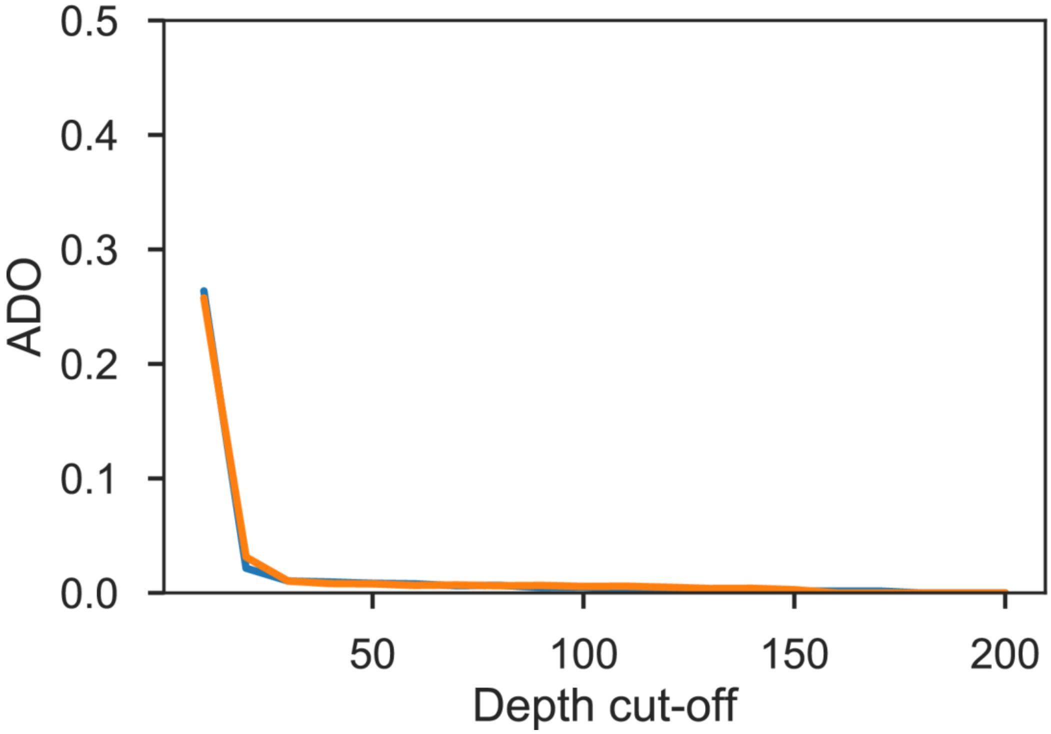

Figure S5. The allele drop-out (ADO) of two unamplified bulk samples after panel enrichment compared to whole genome sequencing data.

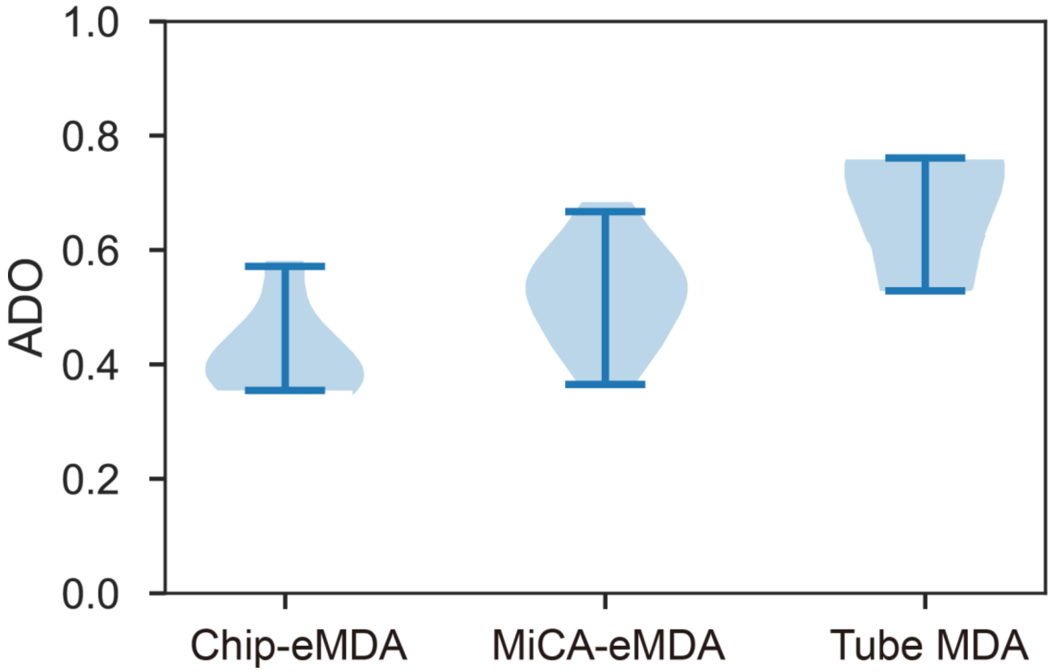

Figure S6. The allele drop-out (ADO) of scWGA products from different methods by deep sequencing after panel enrichment.

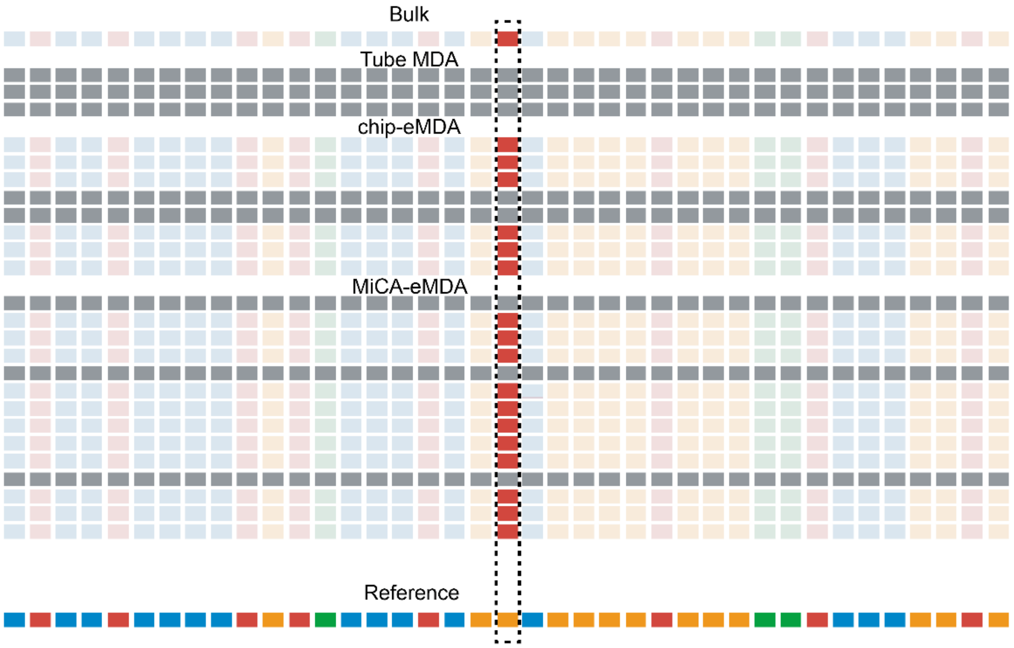

Figure S7. Base composition of individual samples in a region with a valid homozygous SNV. Grey blocks represent loci with no sequencing coverage.

Table S1. The qPCR primers used for quality control

primer set	chromosome	Sequence (5'-3')
#1	1	forward: TTTGATGGAGAAATCCGAGG<br>reverse: CTGACTCGGAGAGCAGGAC
#2	3	forward: AGGCTGCTTGACACTTTGAGGA<br>reverse: TAGCATTGAAGGTGTGCCTTGC
#3	5	forward: CTTGCACCAGAATTGCACTGAA<br>reverse: GATGTCAATTCTCCCCAGACTGA
#4	10	forward: CTTCCTGACCTGTTTGCAGT<br>reverse: CTTCAGTGCACAGAATGCAG
#5	12	forward: CGCTCCTGCCCTTACCTCTATC<br>reverse: AAACCCGGGAGAAGGAGTATCA
#6	22	forward: CTGCCAGCCCAATGTTTGTACT<br>reverse: GGAAGGAAATGAGGCTTCAACC

Table S2. Sequencing data summary of all the samples

sample Name	whole genome sequencing data(G)	panel enriched sequencing data(G)
bulk_1	146.3	3.8
bulk_2	1.4	5.6
chip-eMDA_1	1.9	3.5
chip-eMDA_2	2.3	2.4
chip-eMDA_3	2.1	3.3
chip-eMDA_4	1.8	2.8
chip-eMDA_5	2.2	2.5
chip-eMDA_6	2.2	3.5
chip-eMDA_7	2.7	3.8
chip-eMDA_8	2.4	3.6
mica-eMDA_100ul_1	1.2	-
mica-eMDA_100ul_2	1	-
mica-eMDA_100ul_3	1.2	-
mica-eMDA_100ul_4	1.1	-
mica-eMDA_100ul_5	1.1	-
mica-eMDA_10ul_1	1.2	-
mica-eMDA_10ul_2	0.9	-
mica-eMDA_10ul_3	0.8	-
mica-eMDA_10ul_4	0.8	-
mica-eMDA_10ul_5	0.8	-
mica-eMDA_10ul_6	0.9	-
mica-eMDA_10ul_7	1.4	-
mica-eMDA_20ul_1	1	-
mica-eMDA_20ul_2	1.3	-
mica-eMDA_20ul_3	1.4	3.7
mica-eMDA_20ul_4	1.2	2.8
mica-eMDA_20ul_5	0.9	-
mica-eMDA_20ul_6	1.1	-
mica-eMDA_30ul_1	1.1	-
mica-eMDA_30ul_10	0.8	4.1
mica-eMDA_30ul_11	1	-
mica-eMDA_30ul_12	1	-
mica-eMDA_30ul_13	1	-
mica-eMDA_30ul_2	1.2	-
mica-eMDA_30ul_3	1.1	3.3

mica-eMDA_30ul_4	1.1	3.5
mica-eMDA_30ul_5	1	3.3
mica-eMDA_30ul_6	1.1	3.3
mica-eMDA_30ul_7	1.1	-
mica-eMDA_30ul_8	1.1	-
mica-eMDA_30ul_9	0.9	3.6
mica-eMDA_40ul_1	0.9	3.3
mica-eMDA_40ul_2	1	2.9
mica-eMDA_40ul_3	1	-
mica-eMDA_40ul_4	1	-
mica-eMDA_40ul_5	144.8	2.9
mica-eMDA_40ul_6	0.9	3.7
mica-eMDA_40ul_7	0.9	-
mica-eMDA_40ul_8	0.8	-
mica-eMDA_40ul_9	1.2	-
mica-eMDA_50ul_1	0.9	-
mica-eMDA_50ul_2	0.9	-
mica-eMDA_50ul_3	1.1	-
mica-eMDA_50ul_4	1.3	-
mica-eMDA_50ul_5	1	-
mica-eMDA_50ul_6	1.2	-
tube_MDA_1	0.9	2.5
tube_MDA_2	1	1.8
tube_MDA_3	0.8	1.8
tube_MDA_4	2.4	-
tube_MDA_5	2.9	-
tube_MDA_6	2.2	-
tube_MDA_7	2.2	-
tube_MDA_8	2.3	-
tube_MDA_9	2.5	-

Table S3. The copy number profiling of HeLa unamplified sample

chromosome	start (absolute position)	end (absolute position)	copy number
1	10,385	29,761,479	2
1	31,086,446	55,003,046	3
1	56,178,924	89,765,081	7
1	90,975,802	119,033,213	5
1	120,326,412	247,506,457	4
2	249,260,607	276,243,614	3
2	277,390,497	284,356,528	4
2	285,520,516	378,027,784	3
2	379,239,725	491,696,612	2
3	492,510,170	548,533,431	3
3	549,685,886	590,133,240	2
3	591,377,717	689,477,857	3
4	690,576,122	880,865,128	2
5	881,638,447	896,892,073	7
5	898,065,953	904,633,187	5
5	905,796,654	914,002,640	6
5	915,189,432	926,482,876	7
5	927,743,130	1,061,688,507	3
6	1,062,690,045	1,077,404,032	5
6	1,078,542,876	1,143,262,493	3
6	1,144,562,036	1,164,282,568	2
6	1,165,448,776	1,182,179,288	3
6	1,183,382,047	1,233,001,066	2
7	1,233,667,184	1,368,551,837	3
7	1,369,691,532	1,375,701,777	4
7	1,377,460,604	1,391,647,275	3
8	1,392,877,344	1,435,629,088	2
8	1,440,062,409	1,520,087,249	3
8	1,521,236,921	1,537,701,111	4
9	1,539,169,699	1,678,914,393	3
10	1,680,441,317	1,806,331,248	3
10	1,807,465,858	1,814,774,439	2
11	1,815,978,694	1,817,610,375	2
11	1,818,890,827	1,822,955,180	4
11	1,824,184,686	1,876,797,619	3
11	1,878,079,295	1,885,723,650	2
11	1,886,899,675	1,897,986,776	3
11	1,899,179,073	1,917,197,030	4

11	1,918,390,795	1,950,127,628	2
12	1,950,976,038	2,005,478,234	4
12	2,006,731,421	2,083,904,678	3
13	2,103,786,916	2,139,818,824	2
13	2,141,005,047	2,199,601,358	3
14	2,220,276,991	2,305,582,831	2
15	2,329,770,718	2,366,937,280	2
15	2,368,100,410	2,409,491,301	3
16	2,409,877,097	2,420,561,029	3
16	2,421,688,401	2,438,373,162	2
16	2,440,560,293	2,499,064,230	3
17	2,500,171,865	2,568,482,914	3
17	2,569,640,094	2,580,224,246	4
18	2,581,551,675	2,589,837,904	3
18	2,591,014,240	2,658,546,123	2
19	2,659,535,160	2,718,382,797	3
20	2,718,633,291	2,751,989,131	2
20	2,753,180,543	2,758,981,786	4
20	2,760,142,735	2,781,078,711	3
21	2,797,024,243	2,829,327,763	3
22	2,847,002,828	2,880,747,920	3
X	2,883,732,744	2,918,531,717	3
X	2,919,707,611	3,035,072,959	2
